# Built to exclude: the law is a super-determinant of racial disparities in addiction treatment

**DOI:** 10.3389/fpsyt.2026.1811329

**Published:** 2026-09-30

**Authors:** Erin Michelle Turner Kerrison

**Affiliations:** School of Social Welfare, University of California, Berkeley, Berkeley, CA, United States

**Keywords:** addiction treatment, carceral health, harm reduction, health equity, medications for opioid use disorder (MOUD), opioid use disorder, public health abolition, super-determinant of health

## Abstract

The United States built an evidence-based substance use treatment system that works, just not for everyone. Fewer than one in five adults who needed treatment in 2024 received any form of care, including medications for opioid use disorder (MOUD), and Black and American Indian/Alaska Native communities died of drug overdose at the highest rates. This article argues that closing the gap rests on recognizing law as a *super-determinant* of population health: not a contextual influence but a constitutive force that creates the categories through which treatment is organized. Drawing on constitutive legal theory, racial formation, Public Health Critical Race Praxis, and administrative violence, it shows how law defines who is eligible for care, who may provide it, which facilities are recognized, and which practices count as treatment, and how each category was forged through a century of racialized drug policy. The article traces these legal categories across four domains — people, providers, settings, and interventions — and demonstrates how they converge to yield racially sorted treatment environments in community and carceral settings alike. It argues that addiction medicine must integrate legal analysis and critical race methodology as core competencies, treat legal and policy reform as treatment strategies, and center the most legally excluded populations as the starting point for equitable care. Drawing on abolitionist frameworks in public health and drug policy, it insists that reforms be evaluated not by whether they expand access within existing categories, but by whether they dismantle the arrangements that produce racialized exclusion in the first place.

## Introduction: when the gap is the point: racial disparities in addiction treatment

A century ago, Congress decided that some drug users deserved medicine and others deserved prison. That decision is still being made today. The 2024 National Survey on Drug Use and Health reports that approximately 48.4 million people aged 12 or older met criteria for a substance use disorder in the past year (1). Of the 52.6 million who needed treatment, only 19.3% received any form of care, and the gap for adults actually widened between 2023 and 2024 (1). Among the roughly 4.8 million people with opioid use disorder, only 2.2 million received medications for opioid use disorder (MOUD)—the most effective available interventions (1). This gap is not racially neutral. In 2024, overdose death rates varied sharply by race and ethnicity, from 4.4 per 100,000 among Asian Americans to 51.6 among American Indian and Alaska Native people; Black Americans died at 33.8 per 100,000, compared to 24.7 among white Americans and 17.0 among Hispanic Americans (2). Recent work has shown that state-level homelessness rates are strongly associated with higher drug overdose mortality, underscoring housing instability as a structural overdose risk factor (3).

The racial patterning of this crisis has a history predating the current moment by more than a century. Black and Latinx communities have been subjected to criminalization for the same substance use behaviors met with therapeutic innovation when they affect white populations (4, 5). The Harrison Narcotics Act of 1914 was lobbied through appeals to white racial anxieties about Chinese opium trade and Black cocaine use (6). The punitive escalation of the late 20th century—mandatory minimums, the crack/powder sentencing disparity, mass incarceration—targeted Black communities while white drug use was managed through medical channels (5, 7). The contemporary opioid crisis’s therapeutic orientation coincided with the crisis becoming visibly white. Four “technologies of whiteness”—neuroscience framing, pharmaceutical design, legislative innovation, and targeted marketing—constructed the crisis as medical rather than criminal (4). The Drug Addiction Treatment Act of 2000, which enabled buprenorphine prescribing in office-based settings, was explicitly designed as an alternative to methadone’s regulatory infrastructure and its association with Black and Brown urban drug use (8). This history is not background. It is the origin story of the legal categories through which the treatment gap is produced.

The addiction medicine literature has long documented this treatment gap, cataloguing stigma, provider reluctance, inadequate reimbursement, and fragmented systems as its drivers, yet these explanations share a critical limitation. They tend to be race-evasive. They describe symptoms of racialized legal order without naming the order itself—who remains untreated, where services exist and where they do not, which models of care are sustained and which wither. A race-conscious analysis demands different tools. As Devin Banks (9) argues in her model of racialized substance use stigma, dominant SUS scholarship reproduces this race-evasiveness by treating “race” as a true variable rather than as a representation of racism, relying on population-based surveys that compare prejudice across racial categories instead of theorizing how anti-Black racism structures stigma itself.

Ford and Airhihenbuwa (10) developed Public Health Critical Race Praxis (PHCRP), insisting that race consciousness, not race neutrality, is the prerequisite for rigorous analysis of health inequities. Bridges, Keel, and Obasogie (11) extended this commitment into the health sciences directly, arguing that race and racism are not incidental to medicine but central to its development — and that critical race methods are therefore a prerequisite, not an ornament, for rigorous health-science inquiry. Bowleg (12) argued that intersectionality must function as a structural framework, not a demographic checklist: health disparities are shaped by interlocking systems of racism, sexism, heterosexism, classism, and ableism operating simultaneously, not by additive “barriers.” Viewed through these frameworks, the treatment gap is not a problem of insufficient reach within a neutral system. It is a product of how the system was built, for whom, and whose exclusion is written into its legal structure.

This article argues that making sense of this order, and building responses capable of transforming it, necessitates reconceptualizing the role of law. Specifically, it develops the idea of law as a super-determinant: not merely a powerful influence on health outcomes, but a constitutive force that creates the categories through which the treatment apparatus is organized — who is recognized as a potential recipient of care, who is authorized to deliver it, which sites count as legitimate settings, and which interventions are financed as “treatment.” These legal categories have never been racially neutral; they were forged through the racial scaffolding of drug policy and continue to operate as mechanisms of racial formation. It draws on legal epidemiology, critical race theory, PHCRP (10, 13), intersectionality as structural analysis (12, 14, 15), and the concept of administrative violence (16) to show why treatment inequity persists and what dismantling it requires. Black people, non-binary and transgender people, disabled people, undocumented and non-citizen immigrants, and aging populations are the starting point — the populations whose experiences make the argument visible and whose care is what reform must deliver.

Law does not cause racism; it carries racism forward, translating structural inequality into the specific rules that determine who can access care and on what terms. This distinction matters for intervention. Racism, capitalism, and other structural forces shape the landscape of addiction treatment — but law is the mechanism through which those forces are operationalized, enforced, and made legible to policy. Scope-of-practice rules, methadone clinic siting requirements, pharmacy dispensing restrictions, and Medicaid eligibility conditions do not emerge from nowhere; they encode historical and ongoing racial hierarchy into the architecture of the treatment system. Barnett and colleagues (17) found that racial disparities in MOUD receipt persisted even among patients with frequent healthcare contact — evidence that proximity to providers is insufficient when the legal structure governing what those providers can offer, to whom, and under what conditions remains racially ordered. Law is where racism goes to work in medicine. It is also, therefore, where medicine must go to work on racism.

### A note on scope

This article foregrounds racial formation as its organizing analytic because the legal categories it examines — coverage, criminalization, provider authorization, intervention recognition — were forged through the specific history of racialized drug policy in the United States. That history is irreducibly racist in its origins and operations, and analyzing it requires a framework adequate to that specificity. This does not diminish the independent and intersecting force of ableism, transphobia, xenophobia, and other systems of domination in structuring the treatment gap. The article engages disability (18, 19), trans exclusion (16), and immigration status (20) as intersecting axes throughout, but it does not offer the sustained, centered analysis each deserves. Those analyses are urgent projects I intend to take up in future work. A parallel boundary applies to the level of law examined: this analysis centers federal statutes as the primary unit because Medicaid, the Controlled Substances Act, the inmate exclusion, the Mental Health Parity and Addiction Equity Act, and the Americans with Disabilities Act are constitutive of the categories within which state regulation, private insurance contracting, and local zoning operate. The private insurance market does not exist outside this federal design. It is organized by it.

## Theoretical foundations: constitutive law, racial formation, and administrative violence

### Law as constitutive: from regulation to category creation

Legal epidemiology—the scientific study of law as a factor in the cause, distribution, and prevention of disease, institutionalized through the Center for Public Health Law Research and its LawAtlas policy surveillance platform—has demonstrated that variation in statutes governing prescribing, coverage, and criminalization is associated with variation in health outcomes (21, 22). This work has built the empirical infrastructure for measuring statutory variation across jurisdictions. But it models law as an independent variable acting on pre-existing arrangements—a powerful influence on health, not a constitutive force that creates the categories through which health care is organized. The present argument builds on that infrastructure while parting from its premise.

Legal institutionalism offers a different premise. Law does not merely regulate arrangements that would otherwise exist; it brings them into being by creating categories—”licensed professional,” “insured individual,” “correctional institution,” “covered benefit”—and assigning rights, duties, and powers to each (23). A person’s status as a Medicaid beneficiary or an opioid treatment program’s status as a dispensing facility is not a natural given. It is a legal artifact.

Hartman (24) demonstrated this constitutive power with unmatched clarity. In *Scenes of Subjection*, she showed that law did not regulate the enslaved but produced the enslaved subject through categories of personhood, property, and consent—and that emancipation reorganized rather than resolved this violence, carrying forward the racial logics of the regime it ostensibly replaced. The legal categories organizing substance use treatment today descend from this same tradition: they constitute subjection while performing care, along racial lines operative since the earliest federal drug legislation. This lineage is not confined to the distant past; the documented history of medical experimentation and differential treatment of Black Americans establishes a continuous record of medicine operating as an instrument of racial sorting rather than a neutral corrective to it (25). Bridges (26) observed this dynamic at work in the contemporary welfare state, demonstrating ethnographically that Medicaid enrollment itself is a site of racialization—eligibility functioning as a moral sorting mechanism that governs the bodies of poor women of color while appearing to administer a neutral benefit.

This article proposes *law as super-determinant* as a conceptual framework distinct from existing structural and fundamental cause accounts. Where fundamental cause theory identifies social conditions that generate health disparities across mechanisms (27), and legal epidemiology measures statutory variation as a predictor of health outcomes (21), the super-determinant framework holds that law is not one determinant among others but the constitutive condition under which all other determinants operate. The distinction is not semantic. It has direct implications for what counts as an adequate intervention.

### The racial architecture of drug policy

American drug policy has consistently medicalized substance use for white populations while criminalizing the same behaviors in communities of color (4, 5). The legal categories of contemporary treatment — “opioid treatment program,” “Drug Addiction Treatment Act (DATA) 2000 waiver,” “inmate exclusion” — did not emerge from race-neutral origins. They crystallized through a century of racial formation: the sociohistorical process by which racial categories are created, inhabited, and transformed through state action (28).

When law confines methadone to opioid treatment programs in urban centers while enabling buprenorphine in suburban office-based practices, it constructs a racialized geography of care whose contours track the communities each medication was designed to serve — and the legislative record of DATA 2000 makes that design a matter of congressional record, not theoretical imputation. The Act was explicitly deliberated as a pathway distinct from methadone’s regulatory infrastructure and its association with Black and Brown urban drug use (4, 8). Category creation is racial formation conducted through legal infrastructure. Classification systems — including data infrastructures, risk assessments, and administrative databases — create racial categories while appearing neutral and technical (29). That methodological warning applies directly here: the legal datasets through which researchers track variation across jurisdictions may themselves encode the racial logics they claim to measure.

### Public health critical race praxis and administrative violence

PHCRP provides the analytical foundation for this kind of inquiry, centering race consciousness and linking analysis to action rather than treating racial disparities as incidental findings (10, 13). It has already been applied to racial disparities in MOUD access, demonstrating its relevance to the clinical questions this article raises.

PHCRP was not designed as a clinical instrument — it was designed as an analytic framework for centering race consciousness in public health inquiry and linking analysis to action (10, 30). It has been applied as an organizing lens for racial disparities in epidemiological research and empirically to racial inequities in palliative care (31, 32). The framework’s value here is not as a measurement tool but as a methodological commitment — one that insists race be treated as a structural rather than biological variable, and that analysis be oriented toward dismantling rather than describing disparity. Applied to substance use treatment, PHCRP reorients the research question: not which populations are underserved within the existing order, but how the system’s legal categories were constructed to generate that underservice in the first place. That reorientation is what this article enacts.

The most lethal forms of state violence against trans people are not spectacular discrimination but routine administrative classifications — welfare intake forms, shelter assignments, prison housing designations — that sort people into categories determining access to survival resources. This is administrative violence: harm inflicted through the ordinary functioning of systems never designed to accommodate the people they govern (16). This concept names precisely what the present article describes. When the inmate exclusion severs Medicaid coverage, when licensing standards recognize abstinence-based programs but not harm reduction, when billing codes reimburse physicians but not peer specialists — these are acts of administrative violence distributed along lines of race, gender, sexuality, disability, and citizenship. The concept also forces a tension this article must engage: whether the goal is to make existing legal categories more inclusive or to transform the systems that produce exclusion.

### The synthesis gap

Addiction treatment literature has not resolved this problem. Bradley and colleagues (33) recently argued that designing treatments to fit existing systems — rather than to disrupt them — may perpetuate the very disparities those treatments claim to address. Prison-based therapeutic communities illustrate the point: when Black participants declined to adopt the “addict” identity central to program completion, they were read as treatment-resistant rather than as evidence that the model was never built for them (34). Kaba (35) is blunt: the system is not broken; it is working as designed. If the legal infrastructure produces racialized exclusion by design, then what clinicians and researchers have called treatment failure is a misnomer.

This reframing does not dismiss existing approaches to the addiction treatment gap. Structural determinants research has documented what correlates with disparate outcomes; legal epidemiology has quantified how specific statutes predict variation in care access (21); health services research has mapped the organizational and relational conditions under which treatments reach — or fail to reach — the people who need them (36, 37). What none of these approaches has done is theorize law as constitutive rather than contextual — as the force that creates the categories of person, provider, setting, and intervention through which the addiction treatment system is organized in the first place.

A reasonable objection is that addiction medicine and public health already recognize structural and social determinants of health (SDOH). But recognizing law as a determinant still leaves it as weather, something clinicians and organizations must navigate. The argument here is that law is geology— it creates the organizations, roles, and eligible populations that social determinants frameworks take as given, and it determines the contours of the terrain on which all of that navigation happens. Standard SDOH frameworks group determinants into five domains: economic stability, education access and quality, health care access and quality, neighborhood and built environment, and social and community context (38). Law has no place in that list as a sixth domain. Law is what defines the boundaries of every domain already on it, deciding who qualifies as economically stable enough to lose assistance, whether a criminal record forecloses access to housing or the neighborhood domain, whether health care access is legally available at all. The super-determinant claim is that law is prior to the structural determinants, not parallel to them. Asking what law does to the addiction treatment system means asking, for each domain, how legal classifications construct the categories that make the treatment gap not incidental but designed.

The argument that law shapes clinical outcomes is not foreign to medicine — it is increasingly central to it. Barnett and colleagues (17) published racial disparities in MOUD receipt in the *New England Journal of Medicine*, finding that gaps persisted despite frequent healthcare contact — a finding that implicates legal structure, not clinical practice, as the primary driver. A recent *JAMA* review of MOUD noted federal regulations confining methadone to clinic-only dispensing as a determinant of treatment access — a legal constraint embedded in a clinical review (39). *The Lancet* regards drug prohibition-to-regulation a public health imperative (40). The question this article raises — whether law is constitutive of the treatment gap rather than incidental to it — is one medicine is already asking. Below I take up that question.

Law is super-determinant of health inputs and outcomes not because it is the largest influence among many, but because it constitutes the field within which all other influences operate. Stigma shapes whether a provider offers buprenorphine — but law determines whether that provider is authorized to prescribe it at all. Organizational culture shapes whether a jail offers treatment — but law determines whether Medicaid will pay for it. Provider reluctance shapes who gets referred to harm reduction — but law determines whether harm reduction is a legal activity in that jurisdiction. In each case, the non-legal factor can move the needle within the space law permits. It cannot move the boundary. That is what makes law constitutive rather than contextual, and that is what “super-determinant” means: not the most powerful variable in a regression, but the variable that sets the range within which all other variables can vary.

## Law as super-determinant: category creation as racial formation

If law is constitutive rather than contextual, and if its categories are inseparable from racial formation, then the question becomes concrete: how, exactly, do legal classifications organize the substance use treatment system, and along what lines do they sort? The answer cuts across four domains—people, providers, settings, and interventions—and in each, the treatment gap is downstream of the classifications themselves.

### People: legal construction of eligibility, exclusion, and disposability

Clinical and public health research typically treats the population that could receive treatment as given and then measures who is reached. In substance use treatment, the boundaries of that population are heavily shaped by legal rules with racial content.

Medicaid expansion status illustrates this most directly. In expansion states, adults with low incomes can in principle receive covered treatment. In the ten states that have not expanded Medicaid as of early 2026, people with the same needs and incomes are legally outside that coverage category (41). This is not a random distribution. Nearly three-quarters of the 1.4 million people in the coverage gap live in three Southern states—Texas, Florida, and Georgia—and 97% live in the South (41). Non-expansion states have disproportionately higher proportions of Black and Latinx residents, and uninsured rates in non-expansion states are nearly twice those in expansion states (42). Medicaid expansion reduced uninsurance most dramatically in historically redlined census tracts, suggesting that the legal boundary between expansion and non-expansion operates along the same geographic lines as earlier forms of racial exclusion (42). The ‘coverage gap’ is not an abstract policy artifact. It is a legally constructed racial geography — eligibility functioning as a moral sorting mechanism that governs the bodies of poor women of color while appearing to administer a neutral benefit (26).

Immigration law creates a second boundary of racial exclusion. The Personal Responsibility and Work Opportunity Reconciliation Act and subsequent Medicaid statutes bar most undocumented immigrants and many recent lawful immigrants from full-scope Medicaid, relegating them to emergency-only care regardless of clinical need (43, 44). People who are present but invisible to state care systems constitute a legally produced population for whom the substance use treatment system does not exist. This is not incidental exclusion. Immigration status is a legal construction that creates populations categorically outside the reach of any treatment system premised on insurance coverage (20).

Criminal legal status adds a third layer. The Medicaid inmate exclusion (42 U.S.C. § 1396d(a)(A)) bars federal matching funds for services delivered to incarcerated people, shifting them from community health systems into carceral health regimes with separate—and typically inferior—obligations and options. Given that Black men are incarcerated at roughly five times the rate of white men, the inmate exclusion operates as a racially patterned coverage exclusion (45). Black and Latinx people in jail and prison are also significantly less likely than white incarcerated people to receive any SUD treatment while confined, and are more likely to be placed in punitive rather than therapeutic programming (46, 47)— a racial sorting the inmate exclusion compounds by severing the coverage that might otherwise fund therapeutic alternatives (34). After release, people must navigate re-enrollment during a period of acute overdose risk, and collateral consequences of conviction—housing bans, employment exclusions, supervision conditions—further constrain who appears and stays in treatment (48).

A fourth category is constituted through federal Indian law. The United States holds a treaty-based trust responsibility to provide health care to tribal nations, discharged primarily through the Indian Health Service (IHS). Yet that obligation is met through a discretionary appropriation that has left IHS chronically underfunded relative to need, producing a treatment infrastructure that exists as a distinct legal category but is systematically starved of resources (49). This is not a gap in coverage so much as a coverage structure built to a lower standard by legal design. The consequences are stark in the domain this article foregrounds: AI/AN communities face among the highest overdose death rates in the country, yet MOUD is least available precisely where the trust obligation should guarantee it (50). The federal category “eligible for IHS care” thus functions less as a guarantee than as containment: it names a population the state is obligated to serve while structuring that service to fail.

These rules do not simply influence access within a fixed population. They create distinct legal categories in relation to the treatment system: people who are continuously insured and visible as beneficiaries, people who are intermittently connected, and people who are structurally outside.

Two further axes of exclusion compound this sorting. Trans and gender-nonconforming people face classification systems that force binary gender designations at intake, deny access to gender-appropriate residential treatment, and subject them to discriminatory enforcement in shelters and harm reduction settings—the administrative violence Spade (16) described. Even ostensibly trans-inclusive carceral reforms operate as new modes of gender control rather than relief, because the classifying apparatus itself enacts the harm (51). The intersection of race and sexuality marks a second axis. The structural barriers — spanning healthcare access, stigma, incarceration, and poverty — that block Black men who have sex with men from HIV testing and prevention services include a documented deficiency of services in correctional facilities, even as incarceration rates in this population run disproportionately high (52).

Disabled people face a parallel erasure — absent from the treatment evidence base not by accident but as a structural feature of a field that begins from populations easier to enroll. Dangerousness and criminality are racist and ableist constructs grafted onto disability and “mental illness” — the criminal legal system manufactures disability while deploying disability as justification for confinement (19, 53). Ben-Moshe (19), for example, showed that deinstitutionalization and mass incarceration are not separate trajectories but interconnected processes of carceral reorganization — a redistribution of confinement, not a liberation. Substance use disorder straddles medicalization and criminalization, and the legal sorting of who gets which track maps onto racial and disability status simultaneously. Morgan’s (54) genealogy of race and disability as co-constructions through American law shows that ableist tropes were incorporated into legal definitions of Blackness from the era of chattel slavery forward — constructing Black people as simultaneously physically capable of brutal labor and mentally deficient, then, later, as pathologically criminal — demonstrating that the medicalization/criminalization binary organizing contemporary treatment tracks has deep roots in a legally produced racial-ableist order.

Aging compounds these exclusions in ways the addiction treatment field system has yet to reckon with. Older adults with substance use disorders are the fastest-growing segment of the treatment-need population—pooled data from the 2021 and 2022 National Surveys on Drug Use and Health found that 7.1 million adults aged 60 or older had a substance use disorder in the past year, and 8.5 million were classified as needing substance use treatment, yet fewer than one in three who needed it received any (55)—yet virtually no programming—community or carceral—is designed for their clinical profiles, which often involve polysubstance use alongside chronic disease management, cognitive changes, polypharmacy, and functional limitations. Legal categories governing treatment were built around a younger archetype: the “addict” of federal policy imagination is working-age, physically robust, and either incarcerable or employable. Older adults aging in place after decades of substance use, or aging out of long carceral sentences into a reentry infrastructure that assumes physical capacity and labor-market readiness, occupy a gap between the categories the system recognizes.

The intersection of age with race, gender, and incarceration history is particularly acute for Black women, who face both the cumulative health toll of weathering (56, 57) and the compressed reentry horizon in which diminishing time forecloses the identity transformations that programming demands (58). For menopausal Black women still incarcerated, the harm goes further: carceral infrastructure designed without their physiology in mind, formulary exclusions that function as de facto bans on hormone therapy, and symptom misclassification that routes physiological events into disciplinary channels — including solitary confinement — constitute a cascade of legal and administrative category failures with foreseeable, preventable consequences that meet the constitutional threshold of deliberate indifference (59). Medicare eligibility, Social Security disability determination, and age-based service thresholds introduce additional legal classifications that may conflict with or duplicate SUD-specific categories, creating administrative tangles that function as de facto exclusion for people too exhausted — or too sick — to navigate them.

### Providers: authorized roles and the racial distribution of therapeutic authority

Law does not merely determine who receives care; it determines who is authorized to provide it, and on what terms. In substance use treatment, who counts as a legitimate provider is a legal determination, and that determination is racially ordered.

The methadone/buprenorphine divide is the clearest example. Under the Controlled Substances Act and its implementing regulations, methadone for opioid use disorder is confined to opioid treatment programs (OTPs)—facilities requiring daily or near-daily attendance, concentrated in urban areas, subject to extensive regulatory oversight, and long associated with Black and Brown communities. Buprenorphine, enabled in office-based settings through DATA 2000, was explicitly designed as an alternative to methadone’s racialized infrastructure (4, 8). The legal framework governing who can prescribe what and where was not incidentally racialized; it was built through racial logics. For example, Black patients diagnosed with opioid use disorder are significantly more likely to receive methadone through OTPs than buprenorphine through office-based practices, while white patients follow the reverse pattern (60, 61). This is a distribution that tracks the legal geography of where each medication can be dispensed. One track channels people into heavily regulated clinics requiring daily attendance. The other offers private, flexible care in a physician’s office. The legal boundary between these tracks is also a racial boundary.

Beyond medications, licensure and billing rules determine whose labor is visible to the reimbursement system. Physicians and certain licensed clinicians occupy legally empowered positions as independent billers. Community health workers and peer recovery specialists—who often have particular skill in engaging people marginalized from formal systems—are unrecognized or inconsistently recognized across states (62). Where their work cannot be billed, their roles remain contingent (63). The result is a provider hierarchy in which relational authority (who can actually reach people) and legal authority (whose services can be reimbursed) are misaligned, and the misalignment maps onto race and class.

Legal recognition of “evidence-based treatment” privileges clinical modalities validated in predominantly white samples, while harm reduction, mutual aid, and culturally grounded practices remain outside the categories a capitalist reimbursement system rewards. This hierarchy reflects the racial architecture of the legal categories themselves, not the relative effectiveness of the approaches (64). Black, Latinx, and Indigenous harm reduction practitioners — whose community-based models demonstrably serve marginalized populations more effectively than standardized clinical approaches — are structurally excluded from the provider hierarchy the reimbursement system recognizes (65).

### Settings: facilities as racialized legal categories

Substance use treatment takes place in settings whose existence, location, and permitted activities are determined by law. Those determinations are racialized.

Zoning and siting decisions shape where treatment can physically exist. Neighborhood opposition to OTPs and residential facilities is frequently expressed through zoning challenges, conditional use permits, or nuisance claims that cap capacity or displace facilities from the areas where need is highest — zoning restrictions that have themselves been characterized as strategies to racialize space (66). Treatment deserts are not natural features of the landscape. They are produced by legal decisions about where facilities can operate.

Carceral settings constitute a parallel treatment environment governed by an entirely different legal regime. The coverage exclusion described above structurally separates carceral health from community systems. There is no uniform federal mandate that carceral facilities provide the full range of evidence-based SUD treatment (67). This gap persists despite the constitutional floor established in *Estelle v. Gamble* (1976), which requires only that deliberate indifference to serious medical needs be avoided, not that care meet community clinical standards. In community settings, MOUD represents the clinical standard — yet as documented above, only 2.2 million of the roughly 4.8 million people with opioid use disorder actually receive it, a gap generated by the legal category interactions this article traces. Behind bars, the same medications face a categorically distinct barrier: they are treated as exceptional or risky not because the clinical evidence differs, but because the governing legal categories differ (68). The inmate exclusion, controlled-substance regulations, and the absence of binding care standards combine to engineer a setting in which the community-side access problem is not merely reproduced but legally compounded. Empirical evidence from studies of treatment delivery in jails confirms this compounding: even when treatment infrastructure exists within jails, organizational and legal factors — not clinical ones — determine who receives care (69).

Harm reduction settings occupy the most precarious legal position. Syringe services programs, supervised consumption sites, and low-threshold drop-in centers exist in legal gray zones or are explicitly prohibited in many jurisdictions (64, 70, 71). That legal marginality is not incidental to their mission of meeting people where they are. It reflects the fact that the populations they serve — people who actively use drugs, who are unhoused, who are undocumented, who cycle through incarceration — are themselves legally precarious. Where harm reduction is illegal, it simply does not appear in formal treatment planning, regardless of need.

### Interventions: legal recognition of treatment as racial sorting

Finally, law defines which practices count as “treatment” in ways that channel resources along racialized tracks.

Netherland and Hansen (4) documented how the medicalized track was constructed for white opioid users while the punitive track was constructed for communities of color. This two-track system is maintained through law. Prior authorization rules, step therapy requirements, counseling mandates, and urine drug screening requirements effectively redefine treatment as contingent on abstinence, even when evidence supports continued medication alongside ongoing substance use (72–74). These requirements constitute systematic violations of the Mental Health Parity and Addiction Equity Act, which prohibits more restrictive treatment limitations on SUD benefits, and of the Americans with Disabilities Act, which prohibits MOUD denial in federally funded settings (75).

Research on prison-based therapeutic communities has shown how these dynamics operate at the level of lived experience. In a study of 300 former prisoners who completed at least 12 months of prison-based therapeutic community programming, white participants were more likely to eventually embrace the “addict” label and speak of privileges and reintegrative support received as a result (34). Black participants were more likely to defy the treatment rhetoric — either failing to complete the program or performing a deficit-based self-narrative without investing in its content. The same intervention produced racially divergent outcomes not because of individual differences in motivation, but because the racialized legal and social categories within which it operated made the “addict” label a protective status for some and a compounding stigma for others. As one Black participant put it, the program “was never designed to help Black people” (34).

The historical lineage of these treatment modalities is itself racialized: from the federal narcotic farms of the 1930s through Narcotics Anonymous through therapeutic communities through contemporary medication-assisted treatment, each successive modality better served white participants while leaving participants of color less prepared for recovery (76). Prison-based therapeutic communities remain among the most widely adopted SUD treatment models behind the walls, despite the racist limitations of the modality documented above.

The legal precarity of harm reduction—detailed above as a setting-level exclusion—operates equally as an intervention-level exclusion. Services that fall outside the legal category of “treatment” cannot draw on standard funding streams, regardless of their evidence base. Organizations respond rationally to this incentive structure, building and sustaining the models the legal infrastructure rewards—models historically designed for and validated on white populations.

### The map is the mechanism: legal categories and racial sorting

Across these four domains, law draws the basic map of the substance use treatment system — defining which people are structurally visible, which professionals hold the keys to care, which places can house services, and which practices are named and financed as treatment. The map is racially ordered. Its contours trace a drug policy apparatus assembled across more than a century of deliberate exclusion (77).

Two implications follow for addiction medicine and clinical practice. First, many familiar barriers — “treatment deserts,” lack of prescribers, fragile low-threshold models, gaps around incarceration and reentry — are downstream of legal category creation rather than purely organizational shortcomings. Addressing them means changing the categories, not just the strategies deployed within them. Second, recognizing law as a super-determinant clarifies which problems are realistically addressable through clinical and organizational effort and which reflect hard legal constraints. Where legal categories are permissive but underused, that work may close gaps. The distinction matters for both research and practice.

The persistence of racial disparities in MOUD receipt after controlling for clinical contact is the strongest available evidence that provider and patient factors, while real, are insufficient explanations. Guerrero et al. (78) documented a bifurcated opioid treatment system in which buprenorphine flows to white, privately insured patients through office-based practices while methadone is channeled to Black and Latinx patients through heavily regulated clinics — a distribution that tracks the legal geography of where each medication can be dispensed, not the preferences or reluctance of individual providers. Miles et al. (79) reported persistently high racial disparities in buprenorphine receipt among Medicare beneficiaries from 2015 to 2019, a period of significant provider expansion. Schuler et al. (80) found that broad waiver policies — the primary federal strategy for expanding the buprenorphine prescriber workforce — were insufficient to close racial gaps in distribution. Moon and colleagues (81) showed that racial and economic segregation predicted restricted buprenorphine dispensing at the pharmacy level, independent of prescriber availability. The pattern across these studies is consistent: provider-enabling policies expand access within the existing legal structure without correcting the structural conditions that sort patients along racial lines before they ever reach a provider. Stigma is real. Provider reluctance is real. But they operate inside a legal field whose boundaries determine who arrives at the clinical encounter in the first place — and on what terms.

## Community and carceral settings: where legal categories converge

No single legal category produces the treatment gap on its own. The gap emerges where categories converge—where coverage exclusion, provider scarcity, facility absence, and intervention restrictions compound on the same populations in the same places. Two treatment settings make this convergence visible: community-based treatment and carceral settings. The boundary between them is itself a legal construction with racial content.

### Community-based treatment: who appears, who is invisible

Consider a person with opioid use disorder living in a non-expansion state, recently released from jail, without lawful immigration status. Three legal categories converge on this person simultaneously: Medicaid non-expansion excludes them by income, the inmate exclusion has already severed any prior coverage, and immigration law bars them from full-scope Medicaid regardless. No single barrier explains their exclusion from treatment. The convergence does. Each category alone narrows who can access care; together, they construct a population for whom the community treatment system functionally does not exist — the compound reality facing millions of people whose legal statuses intersect at the seams the system was never designed to cover (20, 82).

Even for people with stable coverage, category interactions constrain what treatment looks like. A Medicaid beneficiary in an expansion state may be eligible for MOUD in principle, but if no opioid treatment program exists in their county—because zoning challenges blocked the only proposed site—and the few primary care clinicians in the area decline to prescribe buprenorphine, the categories of eligible person, authorized provider, and recognized setting fail to align. Coverage without a proximate prescriber in a billable facility is a legal entitlement with no material expression. The “treatment desert” that results is not a single-cause problem; it emerges at the intersection of coverage rules, controlled-substance regulations, scope-of-practice law, and local land use decisions. In rural contexts, these converging legal constraints compound with policing patterns that themselves predict overdose risk, producing circumstances in which the conditions for treatment are legally foreclosed before organizational strategies can operate (83).

The definition of what counts as treatment introduces a further layer of sorting. When abstinence-oriented residential programs satisfy licensing standards and payer contracts more readily than integrated, medication-based outpatient care, organizations build what the legal incentive structure rewards. A person seeking low-threshold, harm-reduction-oriented MOUD may find that the only reimbursable option in their area is an abstinence-mandated residential program—a mismatch not of individual preference but of how law structures the available options. For populations already navigating intersectional disadvantage — trans people confronting binary intake classifications (84), undocumented immigrants afraid to present identification at enrollment, older adults whose needs fall outside programming designed for younger cohorts — the compound effect is not merely reduced access but active repulsion from the system.

Community treatment is not a single entity with uniform reach. It is a patchwork of overlapping legal jurisdictions that produces radically different treatment conditions depending on which categories a given person occupies. Clinical approaches built for the well-insured, residentially stable patient can be effective for that individual. They cannot reach the people living in the gaps between categories — and those gaps are where racial exclusion accumulates and compounds by design.

### Carceral settings: the legal production of exclusion

The community/carceral boundary is not a neutral jurisdictional line. It is a legal switch that flips multiple categories at once. When a person enters jail or prison, the Medicaid inmate exclusion severs their coverage. Controlled-substance regulations make the same medications that were standard of care in the community difficult or impossible to continue. The facility’s licensing and contractual framework determines whether any SUD treatment is offered at all. And the person’s legal status shifts from patient—however tenuously—to inmate, a category governed by constitutional minimum standards rather than clinical best practices (85). These category shifts happen simultaneously, and their interaction is what produces the carceral treatment environment.

James (86) insisted that state violence operates not as aberration but as structural norm—organized at the convergence of race and gender, sustained through institutional routines, and rendered invisible by the very frameworks that claim to oppose it. The carceral treatment environment enacts this insight. The violence is not only the absence of medications; it is the legal regime that defines incarcerated people as categorically different subjects for whom a lesser standard of care is permissible—subjects whose treatment, when it exists, is filtered through security logics that override clinical judgment. The state positions itself as both the source of harm and the arbiter of remedy, offering “rehabilitation” on terms that deepen the subjection of the people it claims to serve.

The interaction between intervention categories and the carceral setting produces its own racialized dynamics. As I demonstrated above, the intervention categories available in carceral settings are overwhelmingly abstinence-oriented therapeutic communities built for a white male archetype. What appears as individual treatment failure is, at the structural level, a predictable output of a custody setting applied to people it was never built to serve.

### The transition as legal rupture

Reentry is not a “gap” in care. It is a legal rupture. Coverage terminates or lapses. Re-enrollment requires documentation, appointments, and administrative processing during a period of acute vulnerability— recently released people face a 129-times higher relative risk of overdose death in the first two weeks after release than the general population (87), a finding replicated across 1.4 million people in eight countries (88). Housing codes, public assistance eligibility rules, and supervision conditions determine whether individuals can stabilize. A person leaving prison may find that the legal categories governing their status—uninsured because coverage has lapsed, ineligible for certain housing because of a conviction, subject to child welfare scrutiny for the same substance use the treatment system claims to address—make sustained treatment difficult even when community programs exist and are willing to engage.

Recent policy developments — discussed more fully in the implications that follow — signal partial recognition of this problem. The Centers for Medicare and Medicaid Services issued guidance in 2023 allowing states to apply for Section 1115 waivers covering pre-release services up to 90 days before release, and as of mid-2025, eleven states had received approval (41, 89). The Reentry Act of 2025, introduced with bipartisan support, would allow states to restore Medicaid up to 30 days before release (90). These are meaningful steps, and a reasonable objection is that they demonstrate the system’s capacity for self-correction—evidence that legal categories are not as fixed as the analysis implies. But these reforms confirm rather than undermine the argument. The X-waiver was eliminated by the Consolidated Appropriations Act of 2023 (91, 92). Reentry coverage required a new waiver authority. Harm reduction authorization required affirmative legislation. In each case, the binding constraint was the legal category itself, and its removal—not organizational improvement alone—was the necessary condition for expanded access. The reforms illustrate that when the treatment gap narrowed, it narrowed because someone changed the law, not because someone ran a better training program within the old rules. In carceral settings, litigation has been among the mechanisms of that change. In *Pesce v. Coppinger* (2018) and *Smith v. Aroostook County* (2019), federal courts held that withholding MOUD from incarcerated people violates the Americans with Disabilities Act (ADA)—which covers substance use disorder as a qualifying disability—and the Eighth Amendment. These cases are establishing an evolving medicolegal precedent that names legal category removal, not organizational willingness, as the necessary condition for access (75). Addiction medicine has an obligation to follow where the courts have led.

That boundary then, is not a natural one. It is a legal construction — maintained by the Medicaid inmate exclusion, by the absence of binding standards for carceral care, by controlled-substance regulations that treat the same medications differently depending on the setting, and by eligibility rules that sever continuity at precisely the moments of greatest risk. And like the other legal categories this article has examined, it is racialized. The populations who bear the full weight of this legal rupture are those rendered what Lisa Marie Cacho (93) termed “socially dead” — stripped of recognizable personhood through interlocking regimes of criminalization, immigration enforcement, and welfare exclusion, disproportionately Black, Latinx, and Indigenous. The community/carceral divide is, in this sense, a technology of racial sorting — one that medicine and public health have rarely named as such.

## What legal literacy makes possible

This section draws out four implications for addiction medicine and health policy. I propose reframing treatment failure as legal design, integrating legal analysis and critical race methodology into clinical and research practice, expanding the repertoire of strategies to include legal and policy change, and centering the most affected populations as the starting point.

The recommendations that follow are not positions in advance of evidence — they are conclusions the evidence has already reached. On decriminalization, studies of Oregon’s Measure 110 — passed through community-organized ballot initiative — found no association between drug possession decriminalization and increased overdose mortality (94–97). Malinowska-Sempruch and Lohman (40) characterized the shift from prohibition to regulation as a public health imperative supported by population-level evidence. On supervised consumption: Milloy et al. (98) documented overdose deaths averted by Vancouver’s supervised injection facility, and Zhu et al. (99) synthesized lessons for North American implementation. On peer specialist reimbursement: Bell et al. (100) conducted a scoping review of peer workforce outcomes, and Gibbons et al. (101) found that peer support was associated with increased buprenorphine receipt and a 0.4 percentage-point reduction in overdose risk among Kentucky Medicaid enrollees. On Medicaid continuity at reentry: Andraka-Christou and colleagues (102) found expert consensus that automatic Medicaid re-enrollment for returning citizens is among the highest-value, most implementable policies available. Each recommendation has a peer-reviewed evidentiary basis. The question is not whether the evidence exists — it is whether medicine is prepared to follow it.

### Reframing failure as design

Much of what medicine calls a ‘barrier’ in substance use treatment is a boundary, simply a line law drew, determining what the system is and who it is for. The absence of MOUD in a rural county is not primarily a function of provider reluctance when the county has no opioid treatment program and no buprenorphine prescriber—conditions determined by licensing, scope-of-practice, and controlled-substance regulations (100, 101). The discontinuity of care at reentry is not primarily an enrollment failure when the Medicaid inmate exclusion has severed coverage by design. The marginality of harm reduction services is not a function of weak organizational capacity when those services are prohibited by state law or excluded from benefit packages.

A race-conscious reframing goes further. It asks not just “what legal rule created this barrier?” but “for whom was this barrier created, and whose exclusion does it produce?” When the legal platform was built through a century of racialized drug policy (4, 5), the racially patterned gap is not an unintended consequence of otherwise neutral rules. It is the foreseeable output of a system whose categories were designed to sort along racial lines.

To be clear, the argument is not that stigma, provider reluctance, and organizational constraints are imagined. They are well-documented and consequential. But they operate within a legally constituted field whose boundaries determine their scope and distribution. Stigma against people who use drugs exists everywhere, but it yields different clinical outcomes in a state that has expanded Medicaid and authorized harm reduction than in one that has not. My principal questions interrogate what structures the conditions under which stigma translates into denial of care—and that structuring is legal. This distinction matters practically. It separates problems addressable by training, technical assistance, and organizational development from problems that rest on hard legal constraints. The legal constraint is not the context for the clinical problem. It is the clinical problem.

Cultural competency training cannot restore Medicaid to someone the inmate exclusion has rendered uninsured. Learning collaboratives cannot create an OTP where zoning law prohibits one. Provider education cannot make harm reduction billable where state law defines it as outside the category of treatment.

### Legal analysis is a clinical competency

The call to expand medicine’s analytic repertoire is not new. Metzl and Hansen’s framework of structural competency urged clinicians to recognize how forces above the individual clinical encounter — policies, economies, and legal regimes — shape health outcomes, and to develop an extra-clinical language for naming them (103). Metzl and Roberts (104) extended this specifically to racism, arguing that structural competency remains incomplete unless it reckons with how medical categories themselves encode racial hierarchy. Legal analysis as a clinical competency builds directly on that call — and names law as the specific structure through which racialized exclusion is operationalized. Emergency physician, Uché Blackstock, has documented how policy rather than biology manufactures racial health inequity, and has called on medicine to treat that fact as a matter of professional responsibility rather than external politics (105). Legal analysis is one form that responsibility takes.

That diagnosis calls for two methodological integrations. First, legal analysis must become a core competency of clinical and public health research, not a peripheral specialty. Systematic mapping of laws across jurisdictions — developed by legal epidemiology (21) — should be combined with PHCRP’s race-conscious orientation (10, 13, 30). Standard legal mapping documents what laws exist. A race-conscious approach goes further: it asks for whom those laws were built, who they exclude, how exclusion is racialized, and how legal categories interact to produce compounded exclusion for populations at the intersection of multiple marginalized statuses. Extending it to the full range of legal categories this article has identified — coverage, immigration status, criminal legal status, provider authorization, facility licensing, intervention recognition — would produce a far more granular account of how law structures the treatment gap along racial lines.

Second, intersectional analysis must function as a structural framework in clinical and public health research, not as a demographic variable added to regression models (12, 14, 15). Studies that examine race, gender, disability, citizenship, and age as separate predictors will miss the compounded exclusion that legal categories produce. The Medicaid inmate exclusion does not operate identically for a Black trans woman with a disability and a white cisgender man; the legal categories converge differently, and the administrative violence (16) is experienced differently because blackness and transness are historically co-constituted rather than separable axes (106). This is why the communities navigating these legal categories must be partners in how those categories are defined and measured, not simply subjects of the analysis.

### Policy change is a treatment strategy

The clinical and public health response to the addiction treatment gap has relied heavily on organizational and individual strategies: training, facilitation, audit and feedback, coalition-building, financial incentives. These are necessary but insufficient when the binding constraint is legal rather than organizational. If law is a super-determinant, then legal and policy change must be recognized as core treatment strategies, not distant contextual influences. This reframing echoes prior calls to put social and policy interventions into clinical practice — including harm-reduction programming in the face of punitive drug laws — as legitimate clinical, not merely advocacy, activity (107).

Scope-of-practice rules should be revised to reimburse peer specialists and community health workers. Licensing and zoning standards should permit integrated, medication-based, harm-reduction models where need is greatest. Medicaid and correctional policy should ensure continuity of coverage through incarceration and reentry. The inmate exclusion should be eliminated. Drug possession should be decriminalized. Harm reduction services — including supervised consumption sites — should be authorized. Each of these addresses a legal category that currently constrains what clinical practice can achieve, and each belongs in the same conversation as prescribing decisions and dosing protocols.

Spade (16) challenges this framing with a question that the article must engage honestly: when the administrative systems themselves are the source of harm, is inclusion sufficient? Easing buprenorphine prescribing rules does not change the fundamental regulatory structure that confines methadone to opioid treatment programs. Expanding Medicaid pre-release coverage does not transform the carceral system that produced the coverage rupture. Kaba (35) Davis (108), and Gossett et al. (109) press the point further. Does expanding treatment within carceral settings legitimize those settings? The abolitionist challenge does not invalidate legal reform, but it insists that reform be evaluated against a different standard—not whether it makes the existing system more inclusive, but whether it moves toward a system world in which the legal categories that produce racialized exclusion are no longer operative. This distinction is one addiction medicine is only beginning to reckon with.

### Centering the most affected populations as starting point

Hooks (110) argued that marginalized communities occupy a position of epistemic privilege — a vantage point from which the operations of power are visible in ways they are not from the center. Ford and Airhihenbuwa (10, 30) operationalized this commitment within PHCRP as “centering in the margins”: beginning analysis from those most affected rather than treating them as a subgroup examined after general findings have been established. This commitment has direct implications for how addiction medicine and public health design studies, select strategies, and evaluate outcomes in substance use treatment.

As discussed in Section III, populations that use substances while occupying the intersection of multiple marginalized statuses — Black men who have sex with men navigating both HIV and incarceration, for instance (52) — remain virtually absent from the treatment evidence base. The field excludes the people most affected by interlocking systems. This is not an oversight. It is a structural feature of a research enterprise that begins from the “general population” and then asks why certain subgroups are underserved. Research that begins instead from the most legally excluded populations — Black people, queer and trans people, disabled people, undocumented and non-citizen immigrants, aging populations — will ask different questions, design different studies, and generate different strategies.

Concretely, this means that equitable treatment research should begin by mapping the legal categories that exclude specific populations, design approaches in partnership with those populations, test those approaches in the settings where those populations actually receive (or are denied) care, and evaluate success by whether the most excluded populations experience measurable improvement — not by whether average outcomes improve while disparities persist or widen.

Methodologically, centering demands changes at every phase of the research process. Sampling strategies should oversample the legally excluded rather than treating them as hard-to-reach subgroups whose absence is noted in a limitations paragraph. Outcome measures should reflect what matters to those populations—sustained housing, reduced criminal legal contact, family reunification, bodily autonomy—not only clinical metrics like abstinence or retention that may themselves reflect the priorities of programming designed without their input. Community advisory structures should hold genuine decision-making authority over research design and dissemination — not serve as consultative ornaments whose sign-off is recorded and then set aside (111, 112).

And, perhaps most importantly, the legal mapping that precedes treatment design should be conducted with the communities it describes, because the people navigating compound legal exclusion possess knowledge about how categories interact that no legal dataset can capture. Recovery programming built for and validated on white male opiate users cannot be adopted wholesale for populations facing intersectional disadvantage. Cultural relevance is a structural question, not a sensitivity training question. It means designing from the margins, not adapting from the center.

## Discussion

The substance use treatment gap is not an implementation problem. It is a legal one. Law constitutes the categories through which the treatment system is organized. Legal categories were forged through a century of racialized drug policy and continue to operate as mechanisms of racial formation, sorting populations into tracks of care and abandonment along lines of race, gender, sexuality, disability, citizenship, and age. Recognizing this reorients, rather than dismisses, the importance of clinical skill, organizational capacity, and provider commitment. It clarifies what those efforts are working against — and what changing the ceiling, not just working beneath it, would require.

I am not claiming law is the only force shaping the treatment gap. I am claiming it is a different kind of force — one that creates the terrain on which every other force moves. They operate within the field it creates, and they cannot move its walls. Stigma, provider reluctance, and organizational capacity operate within a legally constituted field whose boundaries they cannot themselves change. Removing legal barriers is a necessary but not sufficient condition for equity — and that necessity is what existing structural determinants frameworks have not named.

The tension this article cannot resolve is worth naming directly. The implications outlined above are largely reformist: eliminating the inmate exclusion, authorizing harm reduction, and restructuring Medicaid continuity through reentry. Wahbi and Beletsky (113) argue that even ostensibly medical alternatives to incarceration — including coercive SUD treatment — extend carceral reach rather than disrupt it, and that genuine alternatives must be centered on liberation, not the dispersal of punitive power into clinical settings. Woodall and Boeri (114) press the point from lived experience, finding that drug courts and treatment-enforcement collaborations function as carceral extensions regardless of their clinical framing. Spade (16), Kaba (35), Davis (108), and James (86) ground this in the structural argument: if the legal categories were designed to exclude, making them more inclusive may extend the reach of the system without transforming the logics that produced exclusion in the first place. These are not reasons to forgo reform — people are dying now. But the harder question must be held alongside the pragmatic one: is the goal to treat better within existing legal categories, or to create legal categories worthy of the people they govern?

The answer is both — but the second question must discipline the first. The standard against which clinical and policy strategies should be evaluated is not whether they improve average outcomes, but whether they dismantle the legal frameworks that produce racialized disparities in who receives care, what kind of care they receive, and whether that care is experienced as healing or as another form of state control. Addiction medicine that begins from the experiences of the most legally excluded populations — and works backward from their needs to the legal and organizational changes required to meet them — will produce different questions, different standards, and different outcomes than the field has generated to date.

## Conclusions and recommendations

First, clinical and public health research should integrate legal-epidemiological measures into treatment outcome frameworks as a matter of standard practice. Legal status — coverage eligibility, criminal legal involvement, immigration status, facility licensing — shapes who reaches care before clinical contact occurs. Outcome studies that do not account for these upstream legal conditions will systematically misattribute treatment failure to individual or organizational factors.

Second, dismantling carceral exclusions from MOUD access is both a clinical imperative and a public health emergency. The evidence that drug poisoning is the leading cause of death in the first week after release from incarceration — with crude mortality rates of 657 per 100,000 person-years in that window, across 1.47 million people in eight countries (88) — is not a finding that calls for better discharge planning. It calls for eliminating the inmate exclusion, mandating evidence-based SUD treatment in carceral facilities, and building Medicaid continuity through the transition — not around it.

Third, harm reduction should be treated as a primary clinical strategy, not a last resort or a legally marginal supplement to abstinence-based care. Where harm reduction is prohibited or excluded from benefit packages, that prohibition is itself the problem requiring legal remedy, not organizational workaround.

Several limitations warrant acknowledgment. This is a conceptual argument, and the causal pathways proposed here await empirical operationalization and testing in clinical addiction settings. Second, the analysis centers the United States legal-racial context; the argument’s applicability elsewhere depends on how legal category creation operates under different political and historical conditions. Third, the independent and intersecting force of ableism, transphobia, and xenophobia in structuring the treatment gap receives less sustained attention here than each deserves. I claim this unfinished work. Resolving it begins with the communities bearing the weight of a treatment system built to exclude them — and demands that addiction medicine, public health, and law follow their lead.

Among people with addiction, drug poisoning is the leading cause of death in the first week after release from incarceration — a mortality rate so extreme it would constitute a mass casualty event in any other clinical context. The legal infrastructure producing that outcome is knowable, nameable, and changeable. Medicine has the standing and the obligation to say so. Doing so requires treating law as a clinical variable, legal reform as a treatment strategy, and the populations most systematically excluded as the starting point rather than the afterthought. Congress decided a century ago that some drug users deserved care and others deserved punishment. That decision still organizes who counts as a patient, who counts as a provider, which sites count as legitimate settings, and which practices count as treatment. Law is the super-determinant that shapes the structural determinants of health, housing, employment, criminal legal involvement, immigration status, and insurance access, that in turn decide who reaches any of those four categories. The work ahead is to specify, domain by domain, which legal changes would actually close the gap, and to test whether closing it changes who lives.

## Data Availability

No original empirical data were generated or analyzed for this article. Further inquiries can be directed to the corresponding author.
